# Recent advances in CRISPR research

**DOI:** 10.1007/s13238-020-00704-y

**Published:** 2020-03-21

**Authors:** Baohui Chen, Yuyu Niu, Haoyi Wang, Kejian Wang, Hui Yang, Wei Li

**Affiliations:** 1 Department of Cell Biology, and Bone Marrow Transplantation Center of the First Affiliated Hospital, Zhejiang University School of Medicine, Hangzhou, 310058 China; 2 Yunnan Key Laboratory of Primate Biomedical Research, Institute of Primate Translational Medicine, Kunming University of Science and Technology, Kunming, 650500 China; 3 State Key Laboratory of Stem Cell and Reproductive Biology, Institute of Zoology, Chinese Academy of Sciences, Beijing, 100101 China; 4 State Key Laboratory of Rice Biology, China National Rice Research Institute, Chinese Academy of Agricultural Sciences, Hangzhou, 310006 China; 5 Institute of Neuroscience, State Key Laboratory of Neuroscience, Key Laboratory of Primate Neurobiology, CAS Center for Excellence in Brain Science and Intelligence Technology, Shanghai Research Center for Brain Science and Brain-Inspired Intelligence, Shanghai Institutes for Biological Sciences, Chinese Academy of Sciences, Shanghai, 200031 China

The clustered regularly interspaced short palindromic repeats (CRISPR) technology has revolutionized life sciences and developed rapidly. Here, we highlight the recent advances in development and application of CRISPR technologies, including the discovery of novel CRISPR systems, CRISPR base editing and imaging, and the applications of CRISPR in plant breeding, animal breeding, disease modeling and biotherapy.

## THE DEVELOPMENT AND DISCOVERY OF NEW CRISPR SYSTEMS

As a cutting-edge biotechnology, the discovery of new CRISPR genome editing tools are always at the heart of the CRISPR research field (Zhang, 2019). A substantial of exciting works have been reported in the past one year. As a new developed type-II Cas9 ortholog, Nm2Cas9 system with compact effector protein size and simple PAM requirement has been harnessed as a promising alternative for genome engineering and gene therapy (Edraki et al., 2018). In parallel, an abundance of Cas12a orthologs showed editing capacity in human cells (Teng et al., 2019). BhCas12b was also engineered as a powerful gene editing tool (Strecker et al., 2019a).

Apart from the existed CRISPR subtypes, many new subtypes of type-V CRISPR system possessing unique characteristics were identified from the metagenome, including Cas12g, Cas12h and Cas12i, some of which were verified as a programable endonuclease to cleave single-stranded DNA (ssDNA), ssRNA or double-stranded DNA (dsDNA) *in vitro* (Yan et al., 2019). CasX, now assigned to Cas12e family (Koonin et al., 2017), was repurposed as an effective genome editing tool in human cells (Liu et al., 2019b). Cas14, which was classified into Cas12f (Makarova et al., 2019), showed genome editing potential in human cells albeit with very low efficiency (Karvelis et al., 2019).Notably, unlike the classic nucleases from Cas12 family, Cas12k was found as a RNA-guided site specific integration system in *E. coli* (Strecker et al., 2019b), providing the potential to make CRISPR tools to produce precise targeted DNA insertion in the mammalian genome.

Except the above mentioned Class-II CRISPR system, the Class-I CRISPR system with multiple effectors has been harnessed to engineer human genome by diverse strategies, either using native nuclease effector for cleavage (Dolan et al., 2019; Morisaka et al., 2019), or using fused FokI domain (Cameron et al., 2019). Importantly, some members of type-I F systems were repurposed as a tool for site-specific DNA integration (Klompe et al., 2019). These studies inspire further explorations in the CRISPR biology that serves as basis for technology development.

## BASE EDITING TECHNOLOGY

Base editors have been widely applied to perform targeted base editing and hold great potential for correcting pathogenetic mutations (Rees and Liu, 2018). Although early evidence suggested that off-target effects of base editing are rare (Komor et al., 2016; Gaudelli et al., 2017; Kim et al., 2017), recent studies have revealed that substantial DNA and RNA off-target edits were induced by base editors (Jin et al., 2019; Zuo et al., 2019). These off-target edits were sgRNA-independent and induced by the fused deaminase (Jin et al., 2019; Zuo et al., 2019). Moreover, researchers have also attempted to reduce off-target effects by engineering deaminases and obtained improved base editors with low RNA off-target edits (Grünewald et al., 2019; Zhou et al., 2019a). These works illustrate examples of how the off-target effects of base editors can be minimized via biological-insight-driven engineering to extend the utility of these powerful gene editing tools for both research and therapeutic applications.

In addition to DNA base editors, RNA base editors have also been developed by fusion of RNA-targeting protein dCas13 to ADAR, which can make directed A-to-I edits in eukaryotic cells (Abudayyeh et al., 2017). Recently, C-to-U editing was proven to be achieved by fusion of dCas13 with evolved ADAR (Abudayyeh et al., 2019). In addition, two studies have reported that targeted A-to-I edits could also be generated by recruiting endogenous ADAR using engineered RNAs (Merkle et al., 2019; Qu et al., 2019). Continued development of improved or newly generated base editing tools with higher efficiency and fidelity is needed to enhance the impact of this technology in the field.

## DEVELOPMENT OF DCAS PLATFORM FOR IMAGING

The dCas system is a versatile platform which has many more applications. Recruiting fluorescent proteins through the dCas9 system enables real-time imaging of genomic loci and chromatin dynamics in native cellular context (Chen et al., 2013; Chen et al., 2016a; Knight et al., 2018; Wu et al., 2019). Initial studies demonstrated that, when illuminating non-repetitive genomic regions, it requires at least 26 sgRNAs to provide sufficient signal for microscopy detection (Chen et al., 2013). Signal amplification with Suntag, tandem split GFP, or tandem RNA-aptamer has been utilized to enhance the labeling efficiency (Tanenbaum et al., 2014; Cheng et al., 2016; Qin et al., 2017; Ye et al., 2017; Chen et al., 2018; Ma et al., 2018). CRISPR-Tag strategy was specifically developed for labeling non-repetitive protein-coding genes with one to four highly efficient sgRNAs (Chen et al., 2018). It is worth to note that combining CRISPR and molecular beacons (MBs) that can undergo fluorescence resonance energy transfer (FRET), termed CRISPR/dual-FRET MB, enables dynamic imaging of non-repetitive genomic elements with as few as three unique sgRNAs (Mao et al., 2019). CRISPR/MB system might represent a promising system for tracking non-repetitive genomic elements. Multicolor CRISPR imaging can be achieved to label numerous genomic loci simultaneously in a single living cell (Chen et al., 2016b; Fu et al., 2016; Ma et al., 2016b; Shao et al., 2016; Wang et al., 2016). A number of groups have used dCas9 imaging systems to track the dynamics of specific genes, regulatory elements (e.g., telomeres, centromeres, enhancers and promoters), or individual chromosomes (Knight et al., 2015; Zhou et al., 2017; Gu et al., 2018). In addition to mammalian cells, CRISPR-based imaging tools have also been applied to label DNA in other species, including yeast, plant and mouse cells (Dreissig et al., 2017; Duan et al., 2018; Xue and Acar, 2018; Han et al., 2019). Besides live-cell DNA tracking, dCas systems, including dCas9 and dCas13, have been engineered to monitor RNA dynamics. dCas9-FP/gRNA requires a PAMer (synthesized oligo) to label targeting RNA, while dCas13-FP/gRNA is capable of labeling both mRNA and non-coding RNA without additional components (Nelles et al., 2016; Abudayyeh et al., 2017; Yang et al., 2019). Organic dye-labeled sgRNAs in complex with dCas proteins (Cas9 or Cas13) enables robust genomic DNA imaging and RNA tracking in living cells including primary cells (Ma et al., 2016a; Wang et al., 2019c). CRISPR imaging systems are crucial for investigating chromatin architectures and transcriptional regulation in healthy and diseased states, would greatly advance our understanding of how genome is spatially organized in the nucleus to coordinate dynamic gene expression.

## CRISPR APPLICATION IN PLANT BREEDING

Heterosis is exploited to produce elite high-yielding crop lines, but beneficial phenotypes will be lost in subsequent generations owing to genetic segregation. Clonal propagation of F_1_ hybrids through seeds would fix heterosis of hybrid crops. Kejian Wang group developed strategy to enable clonal asexual propagation of hybrid rice by multiplex genome editing of four genes (*REC8*, *PAIR1*, *OSD1* and *MTL*) involved in meiosis and fertilization (Wang et al., 2019b). Venkatesan Sundaresan group also obtained similar result by multiplex editing *REC8*, *PAIR1*, *OSD1* in transgenic rice expressing *BBM1* in egg cells (Khanday et al., 2019).

Doubled haploid (DH) technology could generate pure inbred lines within two generations, substantially accelerates crop breeding process. Shaojian Chen group demonstrated that *in vivo* haploid induction system could be extended from maize to hexaploid wheat by knocking out *MTL*/*PLA1*/*NLD* gene using CRISPR/Cas9 (Liu et al., 2019a). Moreover, they discovered that mutation of *ZmDMP* could enhance and trigger haploid induction in maize, which was further verified to get haploid by CRISPR-Cas9-mediated knockout experiments (Zhong et al., 2019).

To solve the problem of difficult genetic transformation in vast majority of crop varieties, Syngenta Company (Kelliher et al., 2019) and Haiyang Wang group (Wang et al., 2019a) independently developed HI-Edit and IMGE (Haploid-Inducer Mediated Genome Editing) to delivery CRISPR/Cas9 cassette by haploid-inducer pollens of maize. Genome-edited DH lines with desired agronomic traits in the elite maize background could be generated within two generations, without the haploid-inducer parental DNA and the editing machinery.

Developing herbicide-tolerant varieties holds great promise for addressing the worsening weed problems in wheat cultivation. Caixia Gao group generated transgene-free wheat germplasms harbouring herbicide tolerance mutations that confer tolerance to sulfonylurea-, imidazolinone- and aryloxyphenoxy propionate-type herbicides by base editing the acetolactate synthase (*ALS*) and acetyl-coenzyme A carboxylase (*ACCase*) genes (Zhang et al., 2019).

Breeding new fruit varieties with architectures and yields suitable for urban farming will be an important part of future agriculture. Zachary B. Lippman group cultivated new vine-like tomato plants into compact, early yielding plants suitable for urban agriculture by modifying three genes for regulator of tomato stem length (*SlER*), rapid flowering (*SP5G*) and precocious growth termination (*SP*) using one-step CRISPR-Cas9 genome editing (Kwon et al., 2019).

## CRISPR APPLICATIONS IN ANIMAL BREEDING AND DISEASE MODELING

As a precise, efficient and faster genome editing tool, CRISPR system is widely used in animal breeding and disease modeling. Combine with gene editing technology, there are several basic ways to generate animal models with gene modification. To create gene edited animals through microinjection of mRNA or RNP into zygote-stage embryos, or use gene edited donor cells as embryonic stem (ES) cells to create gene edited animals. Using microcarriers like viral vectors and nano-particles to deliver genome-editing components is an efficient way to target specific organs of animals (Amoasii et al., 2018; Gao et al., 2018; Nelson et al., 2019).

Different modeling methods were performed according to the different of species. In rodents or other small animals with shorter reproduction cycles, can produce chimera gene edited animals by ES cells injection, and then obtain homozygous mutated animals through natural mating. But for large animals, the most efficient way to get gene-edited animal is by directly injection of CRISPR system into zygote and embryo transfer performed later. As the improving of efficiency and specificity of CRISPR system, it is increasingly being used in large animals like monkeys (Zhang et al., 2018; Qiu et al., 2019; Zhou et al., 2019b). But one of the drawbacks with CRISPR system is that the same gRNA usually causes various genome types between cells and individuals, so it will be hard to obtained “identical” animal models for one disease mutation.

The breakthrough of clone monkeys research brought a new approach to generate animal models by combining somatic cell nuclear transfer (SCNT) with genome editing technology (Liu et al., 2018). Using the edited cells with the same genome typing as donor cells, the clone animals will have the same genetic background. Through this way, Huntington’s disease pigs and a BMAL1-ablation macaque have been established in recent researches (Yan et al., 2018; Qiu et al., 2019). With the improving of SCNT, such an approach will be the most efficient way to generating large animal models of human diseases, including non-human primate models.

Along with the develop of CRISPR technology, a new technology named as prime editing has emerged, which allows make a few bases replace, delete and insert without creating double-stranded DNA breaks (DSB), the system increased types of genetic mutations (Anzalone et al., 2019). It will be the next generation gene editing tool in animal breeding and disease modeling.

## CRISPR APPLICATION IN BIOTHERAPY

CRISPR-based therapies have been in actively development in many laboratories. There have been too many pre-clinical studies ongoing to be covered in this short section, and therefore we will only cover the notable clinical investigations.

Gene editing therapies can be divided into two large categories: *ex vivo* and *in vivo*. For cells that can be harvested from patients, manipulated in the lab, and then engrafted back into patients, *ex vivo* gene editing is favorable to achieve good efficiency and safety. Correcting genetic mutations in human hematopoietic stem and progenitor cells (HSPCs) is a promising strategy to treat various genetic diseases of hematopoietic system. In particular, disrupting erythroid enhancer of human *BCL11A* induced fetal haemoglobin production, providing a promising strategy for treating β-thalassemia and sickle cell disease (PMID: 26375006). Two clinical studies based on this principle were initiated in late 2018 by Vertex pharmaceuticals. In these phase 1/2 studies, they use spCas9 to modify the erythroid lineage-specific enhancer of *BCL11A* in autologous CD34^+^ HSPCs (Vertex, 2018a, b). While these trails are ongoing, a Chinese team led by Hongkui Deng and Hu Chen published their first case in clinical study using CRISPR-based gene editing (PMID: 31509667). In this study, they edited the HSPCs from an HLA matched donor using spCas9 and transplanted CRISPR-edited HSPCs into a patient with HIV-1 infection and acute lymphoblastic leukemia. This study proved the feasibility and relative safety of the strategy of editing CD34^+^ HSPCs.

In addition to HSPCs, enhancing T cell therapy using CRISPR is also in active development. One phase 1 clinical trials led by a team from Chinese PLA General Hospital is assessing the safety and efficacy of CAR-T cells with *PDCD1* being knocked out by CRISPR (Chinese PLA General Hospital, 2018). Recently, Carl June’s group published their study of TCR-T cells with multiplex gene editing of *PDCD1*, *TRAC* and *TRBC* in three patients with refractory cancer. The long-term engraftment and minimal adverse effect demonstrated the feasibility of this strategy (PMID: 32029687).

In contrast to the above-mentioned *ex vivo* strategies, *in vivo* gene editing therapy is more challenging, due to the requirement of efficient and tissue-specific *in vivo* delivery methods. AAV viral vector is the most popular option, however recent studies showed the high frequency of vector insertion into the cellular genome (PMID: 30778238), raising safety concerns. In addition, the potential immunogenicity of SpCas9 and SaCas9 (PMID: 30692695) need to be considered for *in vivo* therapy. The most advanced clinical trial of CRISPR *in vivo* therapy was based on a preclinical study done by Editas (PMID: 30664785), which showed efficient removal of a disease causing mutation within the intron 26 of *CEP290* gene using AAV-saCas9, in both murine and primate models. Subsequently Allergan, in collaboration with Editas, initiated a clinical trial treating patients carrying a homozygous or compound heterozygous mutation (c.2991 + 1655A > G in intron 26 of the *CEP290* gene) (Allergan 2019) using subretinal injection of AAV vector.

In conclusion, we have seen CRISPR therapy entered the clinical studies in the past year, and many more will come in the following years. With more clinical data accumulate, the efficacy and safety of CRISPR based therapy will be better evaluated, and the best cure for many diseases will be found.
